# How can healthcare professionals better support family caregivers in the final days of life: Could the “Family’s Voice Diary” help? A qualitative study based in an area of high socio-economic deprivation

**DOI:** 10.1177/26323524251340707

**Published:** 2025-05-23

**Authors:** Donna Wakefield, Zoe Booth, Michaela Fay, Matthew Breckons

**Affiliations:** 1 North Tees & Hartlepool NHS Foundation Trust, Stockton-On-Tees, UK; 2 Population Health Sciences Institute, Faculty of Medical Sciences, Newcastle University, UK; 3 Wolfson Palliative Care Research Centre, Hull York Medical School, University of Hull, UK; 4 MF Research Consultancy, Newcastle-upon-Tyne, UK

**Keywords:** end-of-life care, palliative care, health communication, caregivers, healthcare professionals, symptom burden, health services research

## Abstract

**Background:**

Family caregivers play a vital role in supporting patients at the end of life, a role that can impact their own well-being. Healthcare professionals may feel unprepared to support caregivers. The Family’s Voice Diary (FVD) was co-developed with bereaved carers and a wide range of healthcare professionals as a tool to improve caregiver support.

**Objectives:**

To understand challenges carers face in receiving support and whether the FVD has the potential to improve this.

**Design:**

Qualitative interviews with thematic analysis.

**Methods:**

Family’s Voice Diaries were provided to hospital, hospice and community nursing teams, to be offered to carers when a patient was in their final days of life. Each diary invited carers to return the diary if they wished to volunteer for an interview and/or consent to analysis of the content of their completed FVD. Adverts were circulated inviting healthcare professionals to interview.

**Results:**

*n* = 23 diaries were returned, which included written reflections and notes used as an aide memoire to discuss with healthcare professionals. Qualitative interviews were conducted with *n* = 6 healthcare professionals and *n* = 1 bereaved carer. Main themes included the carer’s reluctance to ask for support and healthcare professionals feeling unprepared to discuss dying. Using the diary as a communication aid to build a collaborative relationship with staff was valued. Barriers to implementation included a lack of understanding of the purpose of the diary.

**Conclusion:**

This study adds to the evidence base that there is a need for further carer support and clearer communication at the end of life. The diary appears to be valued as an optional addition to prompt communication, aid self-reflection and signpost to further support. Clearer instruction/training on the purpose of the diary could improve its implementation. Further training for healthcare staff, to enable them to feel more comfortable discussing dying with carers and be able to offer support, would be beneficial.

## Introduction

Family caregivers (either biological or chosen family) play a vital role in physical, emotional and psychological support of patients at the end of life.^1^ It is difficult to know the exact number of family carers; however, previous estimates have suggested that of the 6.4 million unpaid family carers in the United Kingdom, approximately 500,000 are caring for someone with a palliative diagnosis.^2^ Caring for someone at the end of life may be rewarding, but it can also be a source of distress.^3,4^ Whilst carers provide support, they are also in a position where they need support themselves. They may feel overwhelmed, sleep deprived and burnt out, impacting their physical health.^5–8^ Research has shown that having someone to talk to and support self-care can lower psychological distress and improve well-being.^9^

Healthcare professionals are accustomed to providing support to their patients but may be unprepared for how best to provide support to family caregivers.^10^ Previous studies have mainly focused on how nurses approach supporting caregivers at home,^11,12^ with less evidence on potential ways to provide support across settings. Half of all deaths occur in hospital,^13^ with patients from socio-economically deprived backgrounds more likely to die in hospital^14^ than those from less deprived backgrounds. Family caregivers in socio-economically deprived areas are also more vulnerable to poor well-being and are more likely to have higher support needs.^15^ Evidence from bereaved carers has shown that the quality of communication in hospitals is poorer than other settings and that a high proportion of complaints in the NHS relate to end-of-life care.^16^ Provision of clear communication with relatives and support should be equitable for caregivers, regardless of setting. Patients often transfer settings in the final phase of life, and so a way of supporting carers that provides continuity across settings is needed. The addition of written communication to verbal communication is beneficial, including details on practical considerations (such as death certification and signposting to bereavement services).^17^

Previous work^10,15^ has identified some key areas of opportunity for clinicians to better support family carers at the end of life, through addressing some of the main burdens they experience. Potentially helpful interventions included promoting excellent communication with family, demonstrating empathy for family emotions and attending to grief and bereavement.^10^

The families of patients within the Intensive Care Unit (ICU) setting may have similar needs (and may overlap) with those receiving end-of-life care, as clear communication is vital, and carers need considerable support. The use of diaries has previously been described within the ICU, not only to help patients with a narrative of their stay but to support relatives. Studies suggest that the use of diaries has the potential to promote psychological wellbeing and is a way for carers to cope with the emotions of supporting a loved one.^18,19^ Diaries also act as a communication tool.^20,21^ It is known that for some carers, writing and reflecting can be empowering, therapeutic and form part of self-care.^22,23^

With bereaved family carers, members of the public and a wide range of healthcare professionals, we co-produced a low-cost, low-burden tool that could be used to promote communication between healthcare professionals and caregiver (details within “Methods” section). The Family’s Voice Diary (FVD) gives space for carers to write and reflect, with signposting to further support, including bereavement support. This tool is designed to move across settings and empower caregivers to make their voice heard when at the bedside in the final days of life.

The objectives of this study were to understand challenges carers face in communicating and receiving support from healthcare professionals when their relative is in the final days of life, and whether a new tool (the FVD), can help improve communication and carer support. In this article, we explore some of the benefits such a tool might have for both healthcare staff and relatives, as well as some of the barriers and challenges associated with implementing and using the diary in different healthcare settings.

## Methods

### Process of developing a carer communication and support tool (FVD)

User-centred design approaches can support better usability of tools used in healthcare settings^28^ and so the content and design of the new version of the FVD, with a new focus, was developed with bereaved relatives, members of the public, a wide range of healthcare professionals and policy makers, outlined in Table 1. Using the methodological framework of co-design,^29^ feedback on previous experiences were sought, with discussion and refining of ideas, resulting in the development of a prototype diary. The diary was redeveloped and refined repeatedly using feedback over a 12-month period, until it was agreed that it was ready for implementation. Some of the content is shown in Figure 2. With the next step in the process, examining how users experience its use in clinical practice, with a view to evolving further in the future based on feedback.

**Table 1.** Co-production of the new Family’s Voice Diary, using a previous tool (see Figure 1) as a starting point.

Who was involved	Number and methods
Bereaved relatives	*n* = 3 interviewed, recorded, transcribed, thematic analysis
Members of the public	*n* = 8 involved in two focus groups, recorded, transcribed, thematic analysis
Healthcare professionals (including nurses (working in the community, hospital and hospital), hospital doctor, hospice doctor, General Practitioner, bereavement officer, paramedics, specialist palliative care doctor and nurse)	*n* = 24 involved in three focus groups, recorded, transcribed, thematic analysis
Policy makers	Additional discussion and feedback from policymakers within the regional ICB, NHS Trust clinical effectiveness team, national PEOLC team at NHS England

**Figure 1.**
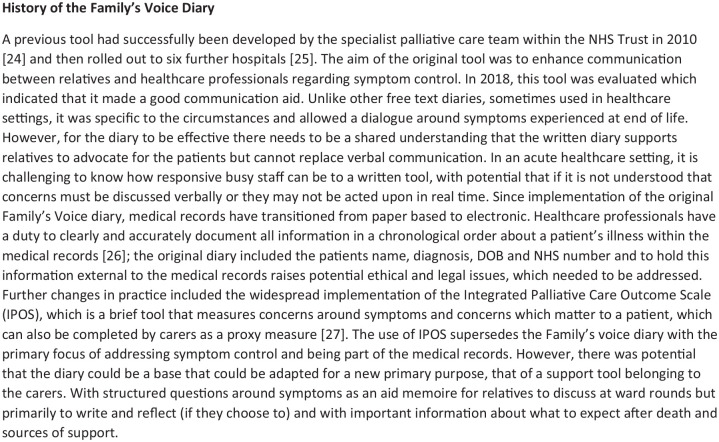
History of a previous tool, which was used as a starting point to develop the new Family’s Voice Diary.

**Figure 2.**
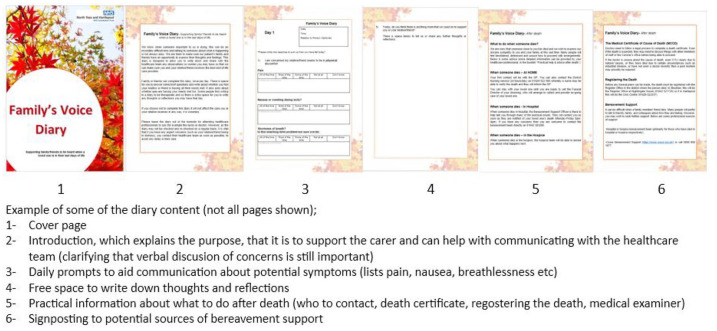
The new Family’s Voice Diary.

The new FVD was printed and distributed to an NHS general hospital (with 563 beds), local District Nurse community hubs and an independent hospice (with 10 inpatient beds) in the north-east of England. Stamped, addressed envelopes were also provided, with instructions to return completed diaries to the study team (unless carers wanted to keep them, in which case it was asked if healthcare professionals could make a photocopy).

The NHS Trusts communication team produced an educational video to raise awareness of the new diary and its purpose, an e-learning package was made available to staff via ESR (Electronic Staff Record learning management for all NHS employees) and regular adverts were sent out to all staff across all settings via the communication and study team.

### Evaluation of the FVD and barriers/facilitators to communicating and supporting carers in the final days of life

#### Summary and analysis of written diary content

The FVD was designed with a final page inviting carers to give consent for the research team to analyse the content of the completed diary, with an additional page provided to add any extra written anonymous feedback. The diaries returned to the team were gathered and held in a secure filing cabinet. The principal investigator (D.W.) then added a summary of the content of each diary to a spreadsheet. The summary was then discussed between the study team and compared and contrasted with the findings of the qualitative interviews.

#### Qualitative interviews

The diaries contained a request that if any relatives were willing to be approached by the research team to discuss a potential short interview in the future should provide their contact details. Those who returned the diary and gave permission to be contacted were then called (by D.W.) to discuss the study (at a minimum of 1 month post-bereavement).

At the request of the study team, the NHS Trust sent out adverts requesting healthcare professionals to volunteer for qualitative interviews about communication and support at the end of life and whether the new FVD was helpful or not, with any barriers/facilitators to its introduction and use.

For both relatives and healthcare professionals, a participant information sheet was provided, and if they agreed to be interviewed, written informed consent was obtained. A convenient time and method (telephone/Teams/face-to-face) was arranged between the participant and interviewer (by M.F.).

Qualitative semi-structured interviews were conducted (by M.F.). Interviews lasted 45–60 minutes. A topic guide was developed to facilitate discussion around general barriers/facilitators in communication and support for carers at the end of life, and also more specific issues relating to the implementation of the new FVD. Interviews allow participants to bring up any additional topics they felt were important. All interviews were conducted via Microsoft Teams (with flexibility offered to be conducted over the phone or in person if preferred), with the audio recording then transcribed verbatim.

D.W. listened to the audio-recordings of all interviews several times to become familiar with the participants’ experiences. M.F. and D.W. read and re-read the transcripts and independently coded the participants’ experiences using an inductive coding approach. Key themes were inductively created independently, using qualitative data analysis software (VERBI) MAXQDA. Authors D.W. and M.F. reviewed the coding, and discrepancies were resolved via discussion to reach a consensus and finalise themes. Illustrative quotes from the interviews were selected and de-identified to be presented within the results.

### Ethical approval

Research Ethics Committee met on 26 June 2023 (East Midland-Nottingham, reference 23/EM/0139, IRAS 319426), with HRA and HCRW approval granted 19 July 2023. Sponsor, North Tees and Hartlepool NHS Foundation Trust.

### PPI involvement

Patient & Public Involvement (PPI) was used in establishing the research question and developing the diary.

## Results

### Summary and analysis of written diary content

A total of *n* = 23 Family’s Voice Diaries were returned with consent given for the content to be analysed and included (anonymously) within this study. Content of the returned diaries is summarised in Table 2. Most returned diaries, *n* = 15/23 (65%), were from the hospital setting. Only *n* = 1 diary (4%) was returned from the community and none from nursing homes, despite extensive efforts to improve participation (frequent reminders to community staff encouraging them to disseminate the diaries and support their return to the study team). Where demographic data was shared (*n* = 7), the diary was used by males (*n* = 4) and females (*n* = 3), ethnicity was mainly white British (*n* = 6) and area-level measure of deprivation was spread (Index of Multiple Deprivation (IMD) 1, 1, 3, 4, 7, 7, 8) including those in the most deprived deciles.

**Table 2.** Summary of content from diaries returned with consent to share.

Place of death	No of diaries returned with permission to share	Demographics (if provided)	Learning points (overall for setting, not just those with demographic details)
Hospital	*n* = 15	Demographics provided *n* = 4/15 Ages: 76, 81, 83, 88 years Gender: 2 female, 2 male Ethnicity: 3 white British, 1 mixed race Black SES (area level measure): IMD: 1, 1, 7, 7 (where 1 is the most deprived and 10 is the least) Cause of death: all non-cancer	• In *n* = 12 diaries, symptom boxes were ticked, and comments were made. • In *n* = 8, there were multiple comments thanking staff for care received, so the perception these relatives had may have been that this is a feedback tool rather than for their benefit/support. • In *n* = 3, the diary was shared between family members who took turns being with the patient and so could pass on what had been happening via the diary. • For *n* = 5 diaries, a relative wrote extensive notes and seemed to understand the reflective purpose of the diary. With one diary used for 19 days.
Home	*n* = 1	Demographics not supplied	• Relative wrote extensive notes and reflections for the patient’s final 14 days of life. Wrote down when they had a seizure and how long it lasted, to tell the district nurse/General Practitioner.
Hospice	*n* = 6	Demographic provided *n* = 3/6 Ages: 66, 75, 85 years Gender: 1 female, 2 male Ethnicity: 3 white British SES (area level): IMD: 3, 8, 4 (where 1 is the most deprived and 10 is the least) Cause of death: all cancer	• One relative found it helpful to use the diary when she changed setting from hospital to hospice. They used the diary to write reflections and even a poem. • As seen in the hospital setting, the diary had feedback about care received and so the perception of purpose was not always clear. • A relative wrote comments about what she had found helpful, such as being able to read about the practicalities of what to do after death included in the diary. • A family wrote in reminders of how best to support their relative for staff to read (as shown in Figure 3).

SES, socio-economic status; IMD; index of multiple deprivation.

**Figure 3.**
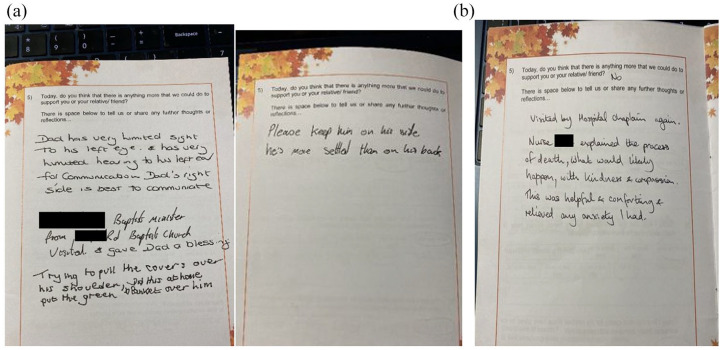
Examples of how the diary was used. (a) To pass on information on how best to care for their relative and (b) what has helped them to feel supported.

Positive uses of the diary included carers using it to write notes and reflect on their experiences (*n* = 6 hospital and community setting), using the diary as a communication aid between each other when multiple family members were visited at different times (*n* = 3) and carers writing reminders to the staff about how their relative likes to be cared for (example shown in Figure 3).

No negative comments were received. However, the number of diaries returned was less than expected, particularly from the community, which may represent a problem in implementation in clinical practice or willingness to return the diary, rather than the specific content. The returned diaries had multiple comments thanking staff for the care received, which may suggest a misperception regarding the role of the diary as a feedback form rather than the purpose being to provide support and improve communication.

Further in-depth analysis of written diary content was not possible due to the limited number of diaries returned (Table 2).

### Qualitative interviews

A total of *n* = 7 semi-structured qualitative interviews were conducted via Microsoft Teams. This included a bereaved carer, hospice doctor, hospital healthcare assistant, hospital-based specialist palliative care nurse, hospital nurse, General Practitioner and community palliative medicine consultant. Themes were grouped into four overarching categories and then into sub-themes, as summarised in Table 3. As participant numbers are small, quotes will be labelled as either a bereaved carer or a healthcare professional, to help protect the anonymity of the participants.

**Table 3.** Key themes identified during qualitative interviews.

Categories	Themes/Subthemes
(1) Challenges identified in communicating and supporting families at the end of life	**(1a) Knowledge of end of life and what to expect** (i) Families do not know what “normal” dying looks like and want more reassurance (ii) Families want more support from healthcare staff, but are reluctant to directly ask (iii) Family members may have different views and support needs **(1b) Healthcare staff end-of-life care skills and confidence** (i) Healthcare staff are hesitant to talk about death (ii) Healthcare staff do not want to intrude
(2) Potential ways that a tool such as the Family’s Voice Diary could help support families and improve communication	**(2a) Improve communication and advocate for the patient** (i) Building a collaborative relationship between healthcare professionals and the family (ii) In a busy acute setting then it is helpful to have communication noted down so it can be acted upon in real time (iii) Helpful when there are multiple family members to open communication with each other **(2b) Help and support benefits** (i) Giving the relatives something to focus on when they are struggling to know how to help (ii) Potential role in pre-bereavement care (iii) Transferring information across services and settings
(3) Implementation barriers	**(3a) Challenges giving out the diary** (i) Knowing when the most appropriate time is (ii) Knowing who is responsible for giving it out **(3b) Structural challenges** (i) Healthcare staff are too busy (ii) Staff turnover/continuity **(3c) The content and style of the diary** (i) Potential to widen inequalities (ii) More self-care advice could be included (iii) Confusion over the primary purpose **(3d) Variation in use in different healthcare settings** (i) The benefits and challenges vary between hospital, home and hospice
(4) Barriers to conducting research/the evaluation process	**(4a) Challenges returning the diary** (i) If the diary is to support the carer, they may not wish to return something personal.

### Challenges identified in communicating and supporting families at the end of life

#### Knowledge of end-of-life and what to expect

##### Families do not know what “normal” dying looks like are reluctant to ask

It is perhaps taken for granted that families know what normal dying looks like. A bereaved relative explained that they were concerned by respiratory secretions (“death rattle”) and if their relative was in pain or distress but struggled to communicate to healthcare staff that they needed reassurance.

Like I wanted to say is this normal? I did say about six hours before he died, “is this normal?” and they just said yes. I said, “Is he in pain?” “I don’t think so.” I wanted someone to say no, he’s not in pain because when you hear that noise you think he’s choking. – Bereaved carer

##### Families want more support from healthcare staff but are reluctant to directly ask

It was acknowledged that caring for someone at the end of life is an emotive time. Relatives felt that healthcare staff did not talk to them as much as they could have, perhaps due to concerns about interrupting.

I understand that they just thought maybe we don’t want to interrupt, L needs to have her last moment with her husband but I did need help and I should have asked. Not for help but reassurance. – Bereaved carer

Especially in an acute hospital setting, which tends to be busier than a hospice setting and also not specifically focused on end-of-life care, staff may be too busy to talk to patients, especially about things that may be perceived as more difficult or time-consuming.

In hospital, a person who is dying, is in a side room and the nurses and doctors are very minimally present. – Healthcare professional

Consequently, the family may want to ask for support but be reluctant to. The diary has the potential to communicate to staff that more support is needed, in a safer way.

I didn’t want to show I was weak by asking for help because I’d been strong all the way through. I was wanting someone to sit with me, but I was frightened to ask . . . . – Bereaved carer

##### Family members may have different views and support needs

In addition, different family members may have differing views on the care needs of their dying relative and perceptions about their discomfort. In these instances, the diary can offer a useful tool not only for relatives to communicate with each other but also for healthcare staff to understand different family members’ views on providing the best possible care for the dying person.

It’s just allowing families to communicate with each other whereby they may not be communicating as well as they want to because they’re tired and they’re hungry and the loved one’s dying and they’ve got all this kind of whirlwind going on, and just to have that as kind of a focus point is really positive. – Healthcare professional

### Healthcare staff’s end-of-life care skills and confidence

#### Healthcare staff are hesitant to talk about death

End-of-life conversations require skills that some healthcare staff may not feel equipped for. Staff may feel uncomfortable and lack confidence talking to relatives about what happens when someone is dying. Whilst this can deter some staff from introducing the diary to relatives for fear of introducing additional upset, it can, conversely, function as a useful bridge or entry point into end-of-life conversations. As one participant put it, in the midst of what is a difficult time for all involved, the diary can be “quite a nice thing to give out.”

Clinical staff will put up as much excuse as possible to engaging with relatives of dying patients. Sometimes they’ll have their own narrative that says, well we don’t want to add to the burden of the relatives. Well, you’re not adding to the burden. It’s your own insecurities, it’s your own unwillingness that inhibits you from going . . . . it’s your own emotional distress. – Healthcare professional

##### Healthcare staff do not want to intrude

Healthcare staff also described feeling hesitant to enter the room when family were present as they were concerned that this was intruding on the family’s privacy.

### Potential ways that a tool such as the FVD could help support families and improve communication

#### Improve communication and advocate for the patient

(i) Building a collaborative relationship between healthcare professionals and the family.
(ii) In a busy acute setting then it is helpful to have communication noted down so it can be acted upon in real-time.
(iii) Helpful when there are multiple family members to open communication with each other.

The diary’s main desired functions are to aid communication and help carers to feel supported. Interviews highlighted ways that it met these aims through supporting families to communicate and advocate for their family member. It was felt that the diary helped to build a more collaborative relationship between healthcare staff and the dying person’s family, helped bridge communication between different family members and increased continuity in the dying person’s care.

The diary . . . it’s a good way to have a collaborative effort between patients and healthcare professionals. So, it’s end-of-life in the hospital, in the hospice, in the community, in care homes. I don’t think the need for it can be overemphasised. – Healthcare professional

It is what it says on the tin. It’s giving people a voice, isn’t it? And it’s giving people the opportunity to write down any concerns that they have. In areas that I’ve worked before, you are very much relying on that family member speaking up . . . if you’ve got somebody who’s more introverted, you’ve got to think about. It’s a little bit less kind of invasive and awkward maybe, and it definitely has its place. – Healthcare professional

Particularly in a busy hospital setting, it was felt to be helpful to have communication noted down so it can be acted upon in real-time.

It (the diary) gives feedback as the situation is happening. So families are empowered to write down their observations. The nurses aren’t in there all the time. The family is often in there for many hours, so they can record things and update the healthcare professionals when they come around to review. – Healthcare professional

In addition to supporting communication between carers and healthcare professionals, the diary was also found to be helpful to facilitate communication between family members themselves, when multiple people accompany a relative at the end of life.

People feel out of their depth and sometimes they feel that their voice isn’t heard. So the fact that we have this is amazing., and often I’ll give it out to families and I’ll go in and to see them, and if it’s found with a really large family, often, they’ve all got different ideas . . . . – Healthcare professional

Some people might be more anxious about a certain thing compared to another family member. So some Family voice diaries that I’ve seen have had multiple family members kind of putting things in, and it’s a way for them to not only kind of communicate the concerns or worries, but its also a way of communicating with each other. – Healthcare professional

#### Help and support benefits

(i) Giving the relatives something to focus on when they are struggling to know how to help.
(ii) Potential role in pre-bereavement care.
(iii) Transferring information across services and settings.

Aside from being a communication tool, the diary has the potential to add focus and purpose to the end-of-life journey. It can give relatives a sense of being useful, having something to focus on at a time when they may be struggling to know how to help.

Some people find it good to use the family voice to record what’s happening. So, that’s how I introduce it to people because often people are sat there and they are twiddling their thumbs, not really knowing what to do . . . it gives them something to focus on and then something to do whilst they sit there for hours and hours watching their relative do very little. It makes them feel maybe more involved. – Healthcare professional

because you’re sitting there kind of, if they’re sleeping, you’re looking at them and you’re thinking, “what can I do?” It would be handy just to have that (the diary) to write notes. – Bereaved carer

Another aspect of providing support to relatives was the potential role the diary plays in pre-bereavement care.

I view it as . . . I always saw it as an episode of bereavement care, pre-emptive bereavement care, believing that if you tell the relative . . . you’re explaining to that relative by those six key areas what is likely to happen in the next day or 24 hours. So, you’re already telling them, start bereaving, start getting involved in the bereavement process. – Healthcare professional

The thing is for these families, they’re going to remember that moment in their lives for the rest of their lives, and I think in palliative care we see so many people and we help so many people and it’s an amazing job. I wouldn’t do anything else, but you do only have that one chance and I would prefer for them to have the Family’s Voice diary and have the option to kind of use it and have that other outlet for communication than not have it. I just think it’s a really, really positive thing. – Healthcare professional

### Implementation barriers

#### Challenges in giving out the diary

(i) Knowing when the most appropriate time is.
(ii) Knowing who is responsible for giving it out.

#### Structural challenges

(i) Healthcare staff are too busy.
(ii) Staff turnover/continuity.

There were multiple barriers to implementing the FVD into routine clinical practice, which primarily related to challenges involved in the process of staff giving out the diary. Some factors echoed the previously discussed issue that healthcare professionals often lack the confidence to discuss dying and struggle to identify the most appropriate time to introduce it to the family.

It’s a confidence issue where the member of staff doesn’t know how to discuss it and how well to explain it to the family? – Healthcare professional

However, it was also reflected that even for healthcare professionals who are experienced at caring for patients at the end of life, prognostication can still at times be challenging and that it is sometimes understandable that healthcare staff are hesitant to express that someone is dying, in case the patient then recovers.

I think it’s quite significant in terms of when a patient is identified as dying. . . . it’s not always straightforward and sometimes you think someone’s dying and they’re not and they recover. I think that is significant in many ways, but one way, obviously with the Family Voice, it might contribute to someone’s hesitancy of giving out things like the Family Voice. – Healthcare professional

Whilst healthcare professionals may be reluctant to state that someone is dying until late in the patient’s trajectory, leading to hesitancy in introducing the FVD until late, a bereaved relative explained that she felt it was given out too late and could have been useful if given sooner.

It wasn’t given to me at the right time . . . if I’d been given that at the beginning I could have written stuff down and it would have helped when he got other visitors, when the family came to visit. They could have had a look at it instead of having to explain this has happened, that has happened. I was getting sick of repeating myself all of the time.

I felt that would have been very helpful to be given it on day one and said, “that is very helpful,” because they can’t be there all the time because they’ve got other people to look at. If you write something down and pop out for a coffee or to the toilet and I come in, I can see what you’ve written. I feel that would have been better. – Bereaved carer

For some, it was not clear whose responsibility it was to give the diary to the family, resulting in nobody taking on the responsibility. This was perhaps understandable due to working in a busy, overstretched healthcare service, where there is high staff turnover and often a lack of staff continuity.

Is it because they don’t have time? Is it that it’s not established whose role it is? I don’t know. Is it the nurse’s role or the doctor’s role on the ward? Do they think it’s a specialist team’s role? I don’t know. – Healthcare professional

#### The content and style of the diary

##### Potential to widen inequalities

It was raised by several participants that the local area is one of the most socio-economically deprived in England and that literacy levels are lower than the national average. Uncertainty over whether relatives can read and/or write was sometimes a barrier to offering the diary, as healthcare professionals did not want to cause embarrassment. Introduction of a tool that is potentially inaccessible to those in need of support could widen inequalities; a simplified/easy-read format was suggested.

Well, they have to be able to read, that rules out, as there are a percentage of my clientele who’s reading age is below six. So, I wouldn’t embarrass people I think . . . . we’ve had a few people recently who the patient themselves can’t read and I am not so sure about the family, so those ones I would rule out. – Healthcare professional

I had one patient . . . . it was only after I’d looked after her for three years that I realised that she couldn’t read because she was so ashamed of the fact. – Healthcare professional

I think it’s in the most deprived area in the country. I realise a lot of people don’t feel comfortable reading. They find lots of text really difficult . . . . we need to err on the side of easy read information . . . . I do think the pages can be simplified using bullet points that people can read and get the key bits. – Healthcare professional

##### More self-care advice could be included

It was suggested that relatives often sit watching their relative and don’t want to leave the bedside. Some details of self-care and what to expect may be a helpful addition for some.

The only thing I wonder is to have a part of the beginning of suggested things that other people have found helpful to do and a little bit on self-care . . . . – Healthcare professional

##### Confusion over the primary purpose

One of the main problems was confusion over the purpose of the diary. In general, people tended to assume that the primary purpose was to give service feedback rather than its actual purpose to support carers and improve communication. This was most evident from the content of diaries returned (Table 2), which suggests that the explanation written within the diary and on introduction by healthcare staff is not clear enough.

I think one thing that people are really used to now is having to make reviews, isn’t it. You order something and you’re immediately asked to review the driver and review the company and review the product and so on. That’s a tick box exercise. That’s how people are thinking I think. – Healthcare professional

If relatives perceived the diary to be a service feedback tool, then they may fear that writing something negative would have consequences for staff and could negatively impact on care received by their relative.

I kind of held back a little bit when I was filling that form in because it was still raw and I didn’t want anybody to get into trouble because there was nothing they could do for him.

The question about, the people who come, do you feel that they’re caring and compassionate, that becomes problematic because if you’re going to put no to one of those, you’re immediately putting yourself in a position where you’re seen to be complaining and a lot of people wouldn’t do that. – Bereaved carer

Conversely, the form could be seen as a “thank you” note to healthcare staff at what is a stressful time. This was also found in the content of diaries returned, many contained messages of gratitude to the healthcare staff.

So when the person who reads the form sees the bit about the after, it’s going to make them happy that what they’re doing there is working . . . . they’re always looking for bits that’s wrong and sometimes it’s really nice to see that something is working. – Healthcare professional

#### Variation in use in different healthcare settings

##### The benefits and challenges vary between hospital, home and hospice

Although the intention was to have one tool which could be embedded into practice across all settings, the challenges and benefits varied depending on the setting; these are summarised in Table 4. Our research suggests that the diary seemed to be of most benefit in the hospital setting, but less so in the community, where patients are in their own home and don’t want to be “medicalised.”

**Table 4.** Variation in the barriers and facilitators of implementing the Family’s Voice Diary in different healthcare settings.

Setting	Implementation challenges/benefits
Hospital	Hospital culture focused on making patients better => reluctance to discuss end of life but also might make relatives feel that care is “too clinical” once their relative is at end of life. The diary can help them feel as though their voice is heard beyond medical intervention. Requires well set-up process and logistics to give out and keep track of the diary, especially if patients are moved out of the hospital into a hospice or community setting. Timing of introducing the diary can be difficult in an acute setting, as often patients die quickly following admission. Can be especially useful, providing a focus and something to do/feel useful for relatives at the bedside when there are no distractions of home life (e.g. in contrast to community). Fewer and more frequently changing staff means the diary can become an anchor for both relatives and staff and bridge communication gaps. Less clear processes and schedules for reviewing patients => useful for relatives to prepare in the interim. Often little communication between the hospital and hospice/community if the patient is discharged/referred.
Hospice	Diary can be seen as a “luxury” in a hospice setting. More staff and closer contact with relatives means that there are generally fewer communication gaps and the diary might have a different function here than in an acute setting (more of a personal aid memoire, than a care tracker).
Community	District nurses are responsible for pain management and often have good insight and a close relationship with relatives. Challenges when multiple district nursing teams and care assistants/agency staff are involved. The diary is considered less important as a communication aid. Potentially useful at the point of General Practitioner involvement/declaring the patient as end of life. Practical considerations of keeping track of the diary are more pronounced in the community.

I think it has been more straightforward offering it in the hospital setting. It has never gone down well in the community and I think there’s lots of reasons for that. So I think in the community, obviously the district nurses pop-in to replace medication syringe driver or to give staff medication, the family there all the time. A lot of patients want to be at home to sort of demedicalise. – Healthcare professional

The whole situation, it has a very different feel to it, if that makes sense. So in the hospital you’ve got, not always, but quite often you’ve got the loved ones sat at the bedside. So then they’ve not got all those home things to be getting on with . . . . So they’re very much focused on the hospital bed in their hospital room, whereas at home they’re getting on with normal life. – Healthcare professional

### Barriers to conducting research/the evaluation process

#### Challenges in returning the diary

For the purposes of this research, we requested families to return their diaries (if willing to do so), which led to a sense of conflict between the purpose of the diary and the research goals. Although the number of diaries returned was lower than expected, making the research on the implementation challenging, if the purpose of the diary is to support the carer and signpost to support, then it is understandable that some would not wish to return it for research purposes.

All the information that we’re giving them within this diary is important. So why would we want to take that away from them? Healthcare professional

Although verbal feedback suggested that relatives had found it helpful,

the thing is I’ve had the verbal feedback from family members who’ve used it, and they found it really helpful, but then obviously they take it away and there’s no formal feedback, it’s really hard to kind of justify the improvement that it’s making. I think it’s really beneficial. – Healthcare professional

I think that’s been kind of really difficult because obviously the families are using it in a really traumatic time of their lives and they may use it to kind of support how they’re feeling and how the family members doing, but then often it’s actually getting that back at the end just so we can learn really. – Healthcare professional

## Discussion

The World Health Organisation defines palliative care as “an approach that improves the quality of life of patients *and their families* who are facing problems associated with life-threatening illness”^30^; yet this holistic approach, which supports not only the patient, but also their caregiver, is not always taken. Whilst healthcare professionals are accustomed to providing care to their patients directly, the well-being of their carers at the bedside may be overlooked. It is known that caring for someone in the final days of life can impact both physical and psychological well-being.^5–9^ Healthcare professionals caring for the patient have the potential to contribute to the wellbeing of carers through clear communication, empathy and signposting to further sources of support if needed. This study found that family carers value support but may be reluctant to ask for it. Whilst some healthcare professionals felt unequipped to discuss death and dying and feared interrupting families at a difficult time.

It is encouraging to see the recent development of tools designed specifically to assess the needs of carers, such as the CSNAT tool, developed to comprehensively assess carer support needs and validated for different conditions.^31,32^ Whilst comprehensive assessments/interventions are beneficial for carers of patients with a palliative diagnosis, these require a skilled and trained practitioner to have such conversations and would ideally be introduced early in a person’s illness. We sought to co-produce and implement a simple, low-cost, low-burden tool, which could facilitate conversations and help carers to feel more supported in any setting, specific to the final days of life and requiring little training to introduce.

Patients living in areas of high socio-economic deprivation are less likely to access healthcare and support, despite having a greater healthcare need^33^; with family carers in deprived areas also reporting worse health and quality of life themselves^34^) In England, there are a higher proportion of unpaid carers in deprived areas compared to least deprived areas, with the highest percentage of unpaid carers in North-East England.^35^ People in deprived areas are also less likely to access specialist palliative care^36^ and hospice care, and in turn leave carers lacking support. Our study was conducted in a socio-economically deprived area in North-East England, where there is an urgent need to find ways to offer carers support at end of life regardless of setting. The FVD is a simple, co-produced tool that is introduced to carers when someone is in the final days of life by any healthcare professional, requiring little explanation. It empowers the carer to make their voice heard and contains signposting to direct them to sources of further support when needed. Our study found that relatives and healthcare professionals felt it was beneficial in facilitating communication between carers, other family members and healthcare professionals, building a collaborative relationship. Some carers found it helpful to write notes and/or and reflect within the diary, which has therapeutic potential, as seen when using carer diaries in the ICU setting.^22,23^ The diaries were felt to form part of pre-bereavement care and also contain sources of bereavement support. Many positive aspects of the diary were identified, however, despite the content being co-produced and intended to be easily readable, it was felt that it could be simplified even further or an easy-read version made for when literacy levels are low, to avoid widening the gap between those with of lower and higher education level. Although the ability to write would also need to be carefully considered, a barrier that may possibly benefit from a digital version of the diary.

### Strengths of this work

How to support carers in the final days of life is an area that warrants further research. Previous studies have mainly focused on carer support in one setting, for specific conditions or are not specific to end-of-life care. We explored barriers in communication and support between healthcare professionals and carers, regardless of setting or cause of death. The feedback was extremely positive, with no concerns of harm through its use. A simple, low-cost tool that does not require specialist knowledge or much time to introduce is something that has the potential to be rolled out more widely, with the potential to improve communication and collaboration between carers and healthcare professionals and provide support and signposting. How the diary was implemented and engaged with varied by healthcare setting. However, the data illustrates that giving relatives the *option* to engage with the diary is the most important thing. Whether or not and how they use it will be as unique as their relative’s end-of-life journey. The value of the diary is therefore not necessarily in how it is being used or it being used in a standardised way, but that it is being offered and explained in ways that are relevant and person-centred.

### Limitations

As previous studies have identified, embedding a new carer support tool can be challenging and requires a proactive approach, with ongoing communication and organisational structures to support its use^37^). The purpose of the FVD is to improve communication between the carer and healthcare professional, yet some healthcare professionals felt uncomfortable starting the conversation about dying and so were less likely to introduce the diary. Further staff training to support staff in introducing and explaining the purpose of the diary would be beneficial, as there was also some confusion due to its previous function. The other purpose of the diary was to have space to write and reflect and to provide support. It was identified that we are explaining that the diary is for the benefit of the carer, which is at odds with requesting it to be returned to use for the purposes of research, as relatives may have written personal notes which they wish to keep. It is also possible that carers decided to keep the diary as it contains signposting such as contact details for bereavement support, or that they were too distressed to return the diary whilst grieving. In part, this may reflect some general challenges to conducting research at end of life, and this limitation meant that we conducted far fewer interviews than planned. The study was conducted without dedicated research time to spend observing the FVD being given to carers, so this study has taken a pragmatic approach and tests how the diary is implemented under real-world conditions without direct observation from the research team. An ethnographic study discussing end-of-life care and how to communicate and support caregivers would shed new light on the situation but was not feasible within this study. A further limitation of the study is that few respondents completed their demographic information, which limited our analysis of diaries returned.

### Further work

A fundamental issue when communicating with and supporting carers is that both the carer and healthcare professionals have multiple goals, and sometimes the pursuit of one goal has the potential to conflict with another goal. For example, carers may feel that their primary goal is caring for the patient and that engaging with a wellbeing tool (where the goal is for themselves to feel supported) may distract them, in conflict with their primary goal. The FVD was created with dual aims so family could contribute to improving the care of their relative (through communicating and recording symptoms) whilst at the same time improving self-care (through reflection and signposting). Previous research has examined multiple goals theory during end-of-life communication.^38^ High-quality communication requires achieving a successful balance of multiple goals, avoiding conflict (which occurs when one goal is pursued at the expense of other goals). Multiple goals theory provides a framework to help balance these multiple goals and has primarily been reported during *verbal* communication at the end of life. However, further evaluation of the FVD could utilise this framework to measure the effectiveness of this intervention, through exploring with carers how they perceive the balance of competing goals when using the diary.

## Conclusion

Caring for someone at the end of life can be an emotive and distressing experience, taking a toll on physical and psychological well-being. Carers need clear communication and support, and although there is no one-size-fits-all approach when it comes to delivering this support, healthcare professionals must remember that providing support to family is part of the holistic and palliative care of their patients. Our study has shown that although the FVD may not be suitable for all carers, for example it would need further development where literacy levels are low, it is a starting point, being structural and flexible enough to enhance communication and help carers to feel more supported when a loved one is in their final days of life. Particularly in areas of high socio-economic deprivation, where carer needs are greater and people are less likely to have accessed specialist palliative care support, a simple low-cost, low-burden tool such as the FVD has the potential to be refined and rolled out further to help give family carers a voice, to feel more supported and have a place in pre-bereavement care.

## Supplemental Material

sj-pdf-1-pcr-10.1177_26323524251340707 – Supplemental material for How can healthcare professionals better support family caregivers in the final days of life: Could the “Family’s Voice Diary” help? A qualitative study based in an area of high socio-economic deprivation Supplemental material, sj-pdf-1-pcr-10.1177_26323524251340707 for How can healthcare professionals better support family caregivers in the final days of life: Could the “Family’s Voice Diary” help? A qualitative study based in an area of high socio-economic deprivation by Donna Wakefield, Zoe Booth, Michaela Fay and Matthew Breckons in Palliative Care and Social Practice
